# APExpose_DE, an air quality exposure dataset for Germany 2010–2019

**DOI:** 10.1038/s41597-021-01068-6

**Published:** 2021-10-28

**Authors:** Alexandre Caseiro, Erika von Schneidemesser

**Affiliations:** Institute for Advanced Sustainability Studies, Potsdam, Germany

**Keywords:** Risk factors, Atmospheric chemistry

## Abstract

Exposure to poor air quality is considered a major influence on the occurrence of cardiovascular and respiratory diseases. Air pollution has also been linked to the severity of the effects of epidemics such as COVID-19 caused by the SARS-CoV-2 virus. Epidemiological studies require datasets of the long-term exposure to air pollution. We present the APExpose_DE dataset, a long-term (2010–2019) dataset providing ambient air pollution metrics at yearly time resolution for NO_2_, NO, O_3_, PM_10_ and PM_2.5_ at the NUTS-3 spatial resolution level for Germany (corresponding to the *Landkreis* or *Kreisfreie Stadt* in Germany, 402 in total).

## Abstract (summary)

**Table:** 

Measurement(s)	air pollution
Technology Type(s)	digital curation • computational modeling technique
Sample Characteristic - Location	Germany

Machine-accessible metadata file describing the reported data: 10.6084/m9.figshare.16680412

## Background & Summary

Air pollution is the largest environmental risk factor for premature mortality. Exposure to air pollution has been clearly linked to the occurrence and severity of cardiovascular and respiratory diseases. The average European loses 2.2 years of life expectancy due to air pollution^1,2^ A number of recent studies have shown that the impact of air pollution on the respiratory system has an adverse influence on the effects of the COVID-19 disease, with particulate air pollution contributing globally to 15 percent of COVID-19 mortality^3–8^.

In order to study the effects of air pollution on human health, exposure datasets are needed. These datasets need to provide comprehensive coverage of air pollutant concentrations over a geographical area and time period. As is often the case, studies that investigate the relationship between air pollution and e.g. health outcomes or social inequalities, produce such a dataset in the context of the study^9,10^ These datasets are rarely published as stand-alone papers and often rely on model data. This makes re-use of such data more difficult. There are exceptions to this, such as the air pollution datasets published by Aaron van Donkelaar and co-workers which incorporate satellite data, modelling, and observational data^11–13^, the latter of which was then used in the Harvard study on the role of air pollution on COVID-19 mortality in the United States^4^. Furthermore, an appropriately high spatial resolution is often critical for such studies.

The dataset presented here was created in the context of a study investigating the role of long-term air pollution in the severity of COVID-19 outcomes for Germany. More specifically, the relationship to COVID-19 mortality, but also additional morbidity endpoints, such as hospitalization and intensive care unit therapy and/or the necessity for mechanical ventilation, were also investigated. In this context we needed a long-term air pollution dataset at the county level for Germany, which we did not find available elsewhere. The air pollutants generally treated in epidemiological studies are particulate matter (PM_2.5_ and PM_10_, particulate matter with an aerodynamic diameter smaller than 2.5 μm and 10 μm, respectively), ozone (O_3_), and nitrogen dioxide (NO_2_). Therefore, in the above mentioned context, we created an air pollution dataset that covers 10 years (2010–2019) at the level of county (for Germany *Landkreis* and *Kreisfreie Stadt*), for PM_2.5_, PM_10_, O_3_, NO_2_, and NO. County level corresponds to the third level of the Nomenclature of Territorial Unit for Statistics (NUTS-3) spatial resolution.

While air pollution monitoring is required in the European Union, as prescribed in the Air Quality Directive^14^, the criteria used to locate monitoring stations (pollution levels, population and availability of funds) often result in heterogeneity in spatial coverage and representativeness^15–17^. Long-term air quality monitoring data is not evenly distributed in space, and furthermore has differing amounts of coverage depending on the air pollutant. At most, only close to one half of the 402 counties had a monitoring station for a given pollutant (Table 1). To provide comprehensive coverage, we combined observational data with model global reanalysis data from the Copernicus Atmospheric Monitoring Service (CAMS), and evaluated a variety of options considering the different types of air quality monitoring data classifications (e.g., urban, rural).

**Table 1:** Yearly NUTS-3 coverage (of a total of 402) by the monitoring network.

Pollutant	minimum coverage	maximum coverage
NO_2_	94	197
NO	121	194
PM_10_	130	188
PM_2:5_	60	110
O_3_	121	197

Such a dataset has a high potential for re-use in different types of health impact assessments, investigation of social inequalities, among other studies. There is also an intention to expand this dataset to provide further coverage for Europe, as well as to refine the use of the CAMS data (e.g. by testing the regional reanalysis besides the global reanalysis, among other possible improvements).

## Methods

In this study, we consider 402 NUTS-3 units for Germany. This reflects the status as of up to November 1, 2016, when the two *Landkreise* Göttingen and Osterode am Harz merged. The sources used for the production of the dataset were Airbase, from the European Environmental Agency^18^ and the CAMS global reanalysis EAC4^19^.

### Airbase

All the data (hourly concentrations of O_3_, NO, NO_2_, PM_2.5_, PM_10_) from air quality monitoring stations located in Germany were accessed between July 11, 2020 and July 20, 2020. Owing to availability, data for the years 2010–2012 were obtained from Airbase-v8, whereas for the years 2012–2018 the E1a dataset was used and for 2019 the E2a dataset was used. Any of the Airbase data used that is not yet ratified, has been flagged, to facilitate avoidance of unratified data if necessary. Airbase classifies the stations based on their type and their siting (called Area in the metadata). Because the focus of the present work is on the long term exposure, the stations of the types “Traffic” and “Industrial” were left out for being considered unrepresentative and those of the type “Background” were included. The background stations thus considered are classified as: rural, rural-nearcity, rural-regional, rural-remote, suburban and urban for Airbase E1a and E2a, and rural, suburban and urban for Airbase-v8.

The following metrics were calculated for each year and for each station where measurements of the pollutant were available and covered at least 80% of hours of the year:

- **NO**_**2**_ annual mean concentration
- number of hours of the year which have a **NO**_**2**_ concentration over 200 μg/m^3^
- **NO** annual mean concentration
- **PM**_**10**_ annual mean concentration
- number of days of the year which have a daily average **PM**_**10**_ concentration over 50 μg/m^3^
- **PM**_**2.5**_ annual mean concentration
- **O**_**3**_ annual mean concentration
- number of days of the year which have a daily average **O**_**3**_ concentration over 120 μg/m^3^
- annual mean of the daily **O**_**3**_ maximum concentration
- maximum daily 1-h average **O**_**3**_ concentration over the entire year
- maximum daily 8-h average **O**_**3**_ concentration over the entire year.

Each station was geo-located within, and each computed yearly value associated to, a NUTS-3 unit. Within each NUTS-3 unit and for each metric, the yearly values per station were averaged in three ways, giving preference, though not exclusiveness, to certain types of stations. Each averaging strategy represents a different scenario:

**average** averaging the yearly values from all the stations within the NUTS-3 unit;

**urban** averaging of the yearly values from stations located at the most urban location types. The location types are, in order of preference: urban, suburban, rural-near city, rural-regional and rural;

**remote** averaging of the yearly values from stations located at the most remote location types. The location types are, in order of preference: rural, rural-regional, rural-near city, suburban and urban.

**Fig. 1:**
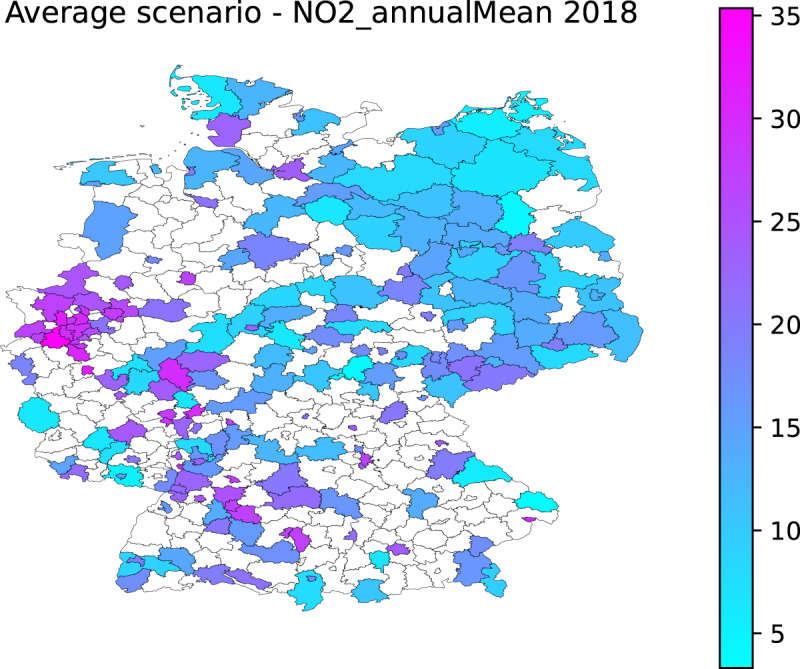
NO_2_ estimated yearly concentration (μg m^−3^) for the average scenario at the NUTS-3 level for 2018.

The methodology described in the previous section produces data for the NUTS-3 units and the years where monitoring data for a given pollutant is available (e.g. Figure 1 for NO_2_ in 2018). Table 1 shows the number of NUTS-3 units covered by data from Airbase. The coverage is always below half of the total number of NUTS-3 units for Germany (maximum of 197 out of 402).

In order to fill any NUTS-3 units missing observational data, we considered three options: (1) use the regional (relative to the *Bundesland*, or NUTS-2) average, (2) use the nearest neighbour and (3) use the CAMS EAC4 global reanalysis^20^, (see next section) after scaling.

Gap filling based on regional averages produced spatial uniformity over large areas (the *Bundesland* or NUTS-2 units), regardless of the nature of the NUTS-3. Using the value from the nearest neighbour overcomes the main drawback of the *Bundesland* approach, but produces artifacts in the form of pairs of NUTS-3 units with different typology (e.g. urban and rural) but equal exposure. Such happens mainly close to large cities: e.g. a rural NUTS-3 unit without any monitoring station adjacent to a large city, that has monitoring stations, ends up, under this strategy, with the same exposure value as the large city.

### CAMS

Due to those limitations we opted to explore the use of CAMS global reanalysis data to do the gap filling.

The CAMS reanalysis was checked for specific regional bias over Germany. The main biases impacting the present dataset for the latitudinal belt 40–50° N in Europe of the product are a ±15% bias for tropospheric ozone, with a seasonal cycle, and an underestimation of wintertime NO_2_ columnar concentrations over part of Europe^21^. We estimate these biases to be either acceptable or compensated, at least partly, by the scaling procedure used (see below). The fields over Germany and for the study period were accessed on September 14, 2020 from the CAMS Atmosphere Data Store.

**Fig. 2:**
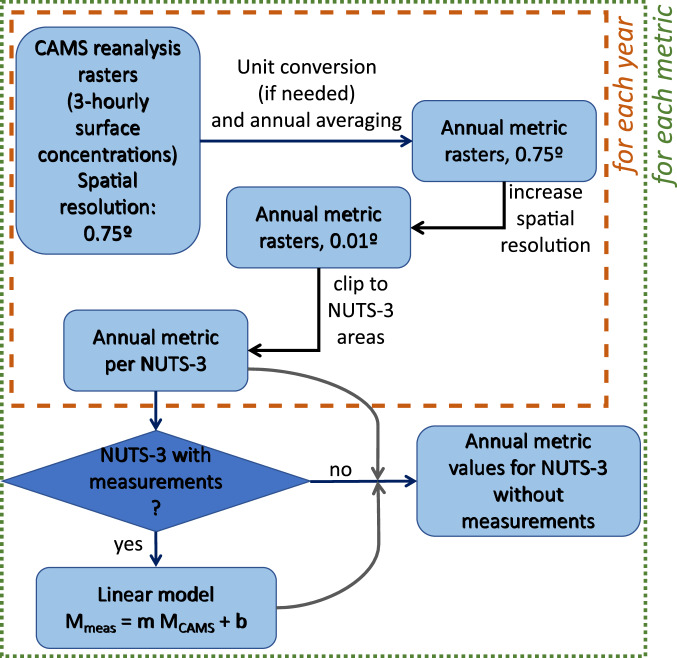
Flowchart for the CAMS data: from the CAMS rasters to the annual metrics for the NUTS-3 units which do not have measurements (or do not have measurements with sufficient spatial coverage) on a given year. The procedure is used for the following metrics: NO_2_ annual mean concentration, NO annual mean concentration, PM_10_ annual mean concentration, number of days of the year which have a daily average PM_10_ concentration over 50 μg m^−3^, PM_2.5_ annual mean concentration, O_3_ annual mean concentration, number of days of the year which have a daily average O_3_ concentration over 120 μg m^−3^.

The procedure followed to compute the CAMS-based metrics is outlined in Fig. 2. Each metric with a time resolution equal to or longer than one day was computed for each cell, after averaging the 3-hourly output timestep to daily values. Those metrics were: NO_2_ annual mean concentration, NO annual mean concentration, PM_10_ annual mean concentration, number of days of the year which have a daily average PM_10_ concentration over 50 μg m^−3^, PM_2.5_ annual mean concentration, O_3_ annual mean concentration, number of days of the year which have a daily average O_3_ concentration over 120 μg m^−3^.

**Fig. 3:**
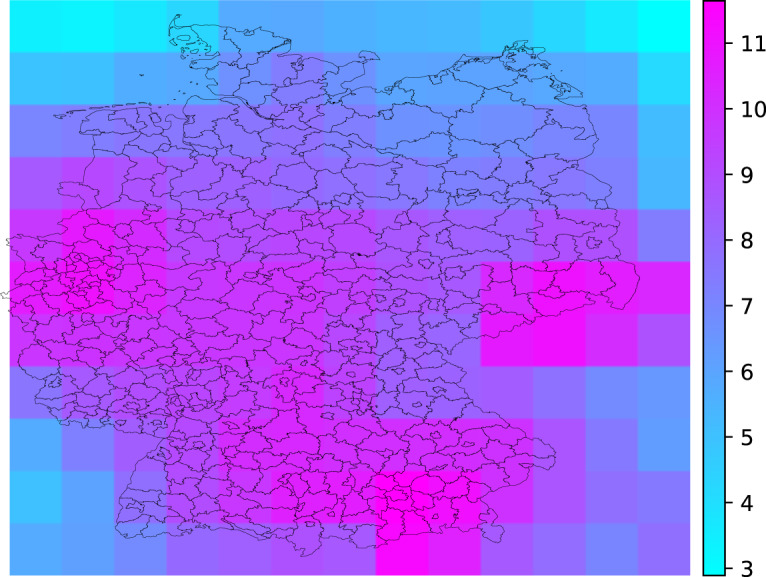
NO_2_ yearly (2018) average concentration (μg m^−3^) from the CAMS EAC4 global reanalysis.

**Fig. 4:**
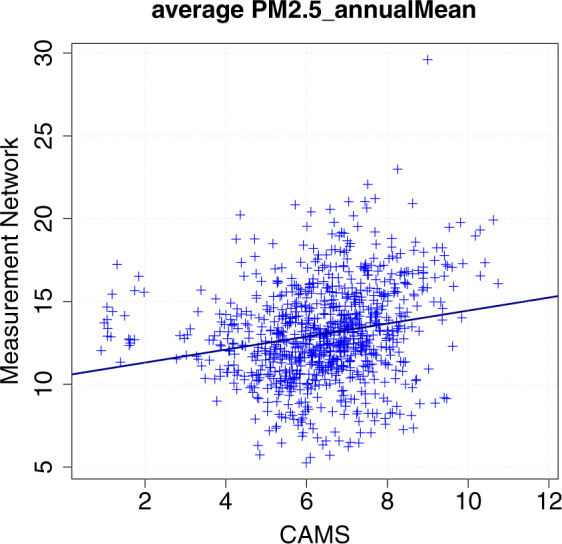
An example of a relationship between an Airbase-derived metric and a CAMS-derived metric (shown here PM_2.5_ annual average) for the NUTS-3 units where monitoring data was available with satisfactory temporal coverage. The linear scaling function thus computed (solid line in the plot) is used to produce the CAMS-derived metrics for the NUTS-3 units where monitoring data was not available with satisfactory temporal coverage.

The resulting rasters (e.g. Figure 3 for the NO2 2018 yearly average concentration) were clipped (area weighted mean) to each NUTS-3 unit area after upscaling the spatial resolution from 0.75° to 0.01° (each smaller cell having the same value as its parent, larger, cell), resulting in verctorized metrics (one value for each NUTS-3 unit, each metric and each year). For each metric and under each scenario a scaling function between the vectorized CAMS values and the respective Airbase-based values was derived. Figure 4 shows the scaling function used to produce, for the NUTS-3 units where monitoring data was not available with satisfactory temporal coverage, CAMS-derived PM_2.5_ from the clipped rasters and the monitoring-based NUTS-3 annual average.

The scaling function was then used to produce CAMS-derived values for the NUTS-3 units and the years where no monitoring data is available or is available with insufficient temporal coverage (Fig. 2).

Despite the considerable amount of scatter between the CAMS-derived data and the monitoring-derived data (e.g. Fig. 4 for PM_2.5_), the approach using CAMS for gap filling overcomes the limitations that arose when using the nearest-neighbour or the *Bundesland* approaches.

**Fig. 5:**
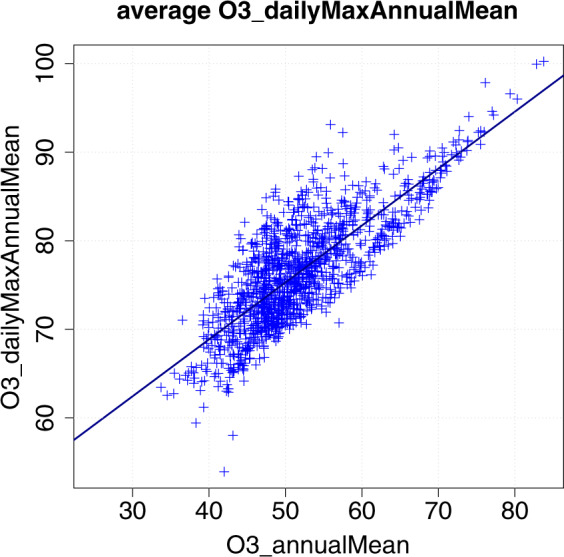
Relationship between the monitoring-based O_3_ daily maximum annual mean and the monitoring-based O_3_ annual mean for the average scenario. The linear function (solid line in the plot) will be used to derive the CAMS-based O_3_ daily maximum annual mean for the NUTS-3 units where monitoring data was not available with satisfactory temporal coverage.

For each scenario, a linear relationship between the metrics with a time resolution shorter than one day and a metric of the same pollutant with a time resolution equal or larger than one day was derived from the Airbase-based values and used with the CAMS-derived values to produce CAMS-based data for those metrics. The number of hours of the year which have a NO_2_ concentration over 200 μg m^−3^ was therefore computed from the NO_2_ annual mean concentration. The annual mean of the daily O_3_ maximum concentration, the maximum daily 1-h average O_3_ concentration over the entire year and the maximum daily 8-h average O_3_ concentration over the entire year were computed from the O_3_ annual mean concentration. An example of such a scaling function is shown in Fig. 5.

## Data Records

**Fig. 6:**
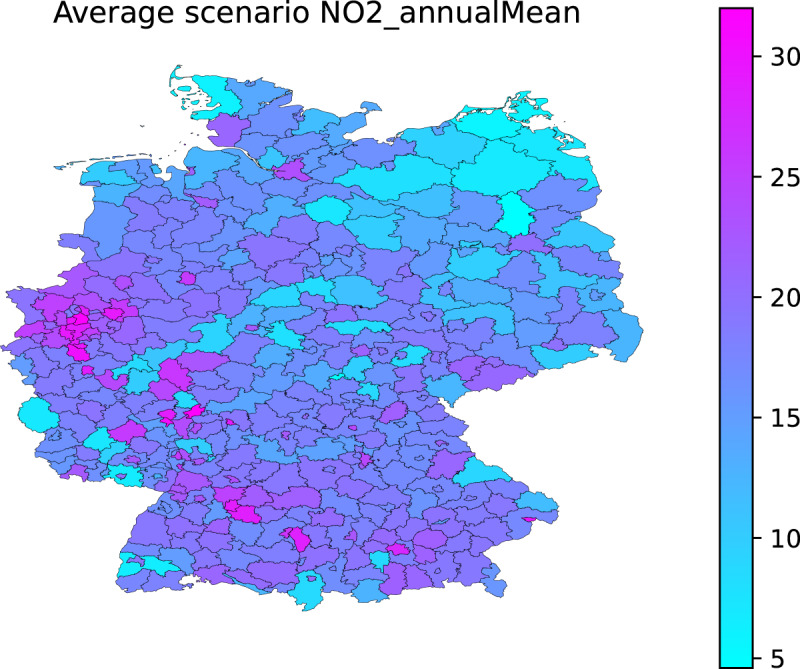
NO_2_ decadal (2010–2019) average concentration (μg m^−3^) from the APExpose_DE dataset.

As a final step, the Airbase and CAMS derived data are combined to produce the APExpose_DE dataset. As an example, Fig. 6 shows the decadal average of the NO_2_ yearly averages.

The APExpose_DE dataset is available in the form of an ASCII file: *APExpose_DE__2010*–*2019.csv*^22^. Each record (each line in the file) corresponds to a NUTS-3 unit (identified by its name and its code), and a scenario, for a given year. There are 402 NUTS-3 units in Germany and 3 scenarios were developed, the total number of records in the dataset is 1206 per year, or 12060 for the entire study period. Each record includes a numeric value for each metric considered.

The ratification status of the Airbase data used for each NUTS-3, year, scenario and metric is given in the file *APExpose_DE__2010*–*2019__Ratified.csv*. The station types used for each NUTS-3, year, scenario and metric are listed in the file *APExpose_DE__2010*–*2019__StationTypes.csv*. These two metadata files have the same structure as the main file.

While we plan to expand the dataset to cover further European countries, this was beyond the current scope of this study. As updates or expansions to the dataset are carried out, these will be noted in the open access dataset.

## Technical Validation

The air quality data used to generate this dataset goes through quality assurance and quality control before being made officially available. In addition, we only used data from sites where 80 percent or more of the hourly data was available, so as to not introduce any seasonal or other bias.

Three different averaging options, corresponding to three scenarios (average, remote and urban), were evaluated for determining the concentration based on monitoring data for those NUTS-3 units where data was available. The comparison of these options showed that while differences did result, they were minor: the 95^*th*^ quantile of the relative difference between the rural or the urban scenario relative to the average scenario was 7.6% and 6.5%, respectively. The different options/scenarios are furthermore provided in the dataset and can be further evaluated and selected based on the use case where they are to be implemented.

## Usage Notes

The ASCII format of the provided dataset enables a simple access and workup. The NUTS-3 code, provided for each record, enables linking the dataset to other, possibly vectorized, datasets at the NUTS-3 or coarser level.

## Data Availability

The code used to generate the dataset can be obtained under the same doi^22^.
